# Racial differences in distribution of fatty acids in prostate cancer and benign prostatic tissues

**DOI:** 10.1186/s12944-019-1130-4

**Published:** 2019-11-03

**Authors:** Xinchun Zhou, Hao Mei, Joshua Agee, Timera Brown, Jinghe Mao

**Affiliations:** 1 Department of Pathology, Cancer Institute University of Mississippi Medical Center, 2500 North State Street, Jackson, MS 39216-4505 USA; 2 Department of Data Science, University of Mississippi Medical Center, Jackson, MS 39216 USA; 3 Department of Biology, Tougaloo College, Tougaloo, MS 39157 USA

**Keywords:** Prostate cancer (PCa), Total fatty acid (TFA), Free fatty acid (FFA), Lipidomics, Racial disparity

## Abstract

**Background:**

It remains controversial whether and which fatty acids are different between prostate cancer (PCa) and benign prostatic tissues (BPT) in association with occurrence, progression and racial disparity between African American (AA) and Caucasian American (CA) populations.

**Methods:**

Total fatty acids (TFA) and free fatty acid (FFA) were determined on fresh frozen prostatic tissues including 26 PCa and 21 BPT from AA and CA patients by Gas chromatography with flame ionization detection (GC-FID) and Electrospray Ionization Mass Spectrometry (ESI-MS), respectively.

**Results:**

In all studied population, TFA in 8 out of 16 individual species, in total and in groups of saturated total fatty acid (STFA), mono-unsaturated total fatty acid (MUTFA), poly-unsaturated total fatty acid (PUTFA) and n-6 TFA were significantly higher in PCa than in BPT; FFA in 4 out of 10 individual species, in total and in groups of MUFFA, PUFFA, n-6 FFA and n-3 FFA were significantly higher in PCa than in BPT. The concentrations of most fatty acid parameters correlated with Gleason’s grade and clinical stage of PCa. As compared with CA men, AA men had higher concentrations of TFA, especially TFA with chains of 14–18 carbons than in BPT, and lower concentrations of TFA in PCa.

**Conclusions:**

Increasing in prostatic fatty acids in the form of TFA and FFA correlated to occurrence, progression and racial disparity of PCa.

## Introduction

Prostate cancer (PCa) threatens men’s health worldwide [1–3]. In US, PCa is the most diagnosed non-skin cancer and second leading cause of cancer deaths [4]. According to The American Cancer Society’s estimates, there will be about 174,650 new cases of and about 31,620 deaths from prostate cancer in US for 2019 (https://www.cancer.org/cancer/prostate-cancer/about/key-statistics.html). While age, family history, and race have been well defined as unmodifiable risk factors for PCa, identification of modifiable risk/protective factors is of great significance in prevention and treatment of PCa. Fatty acids are most disputed among modifiable factors.

PCa is known to be unique in its energy metabolism: while cancer cells in majority of malignancies exhibit increased glycolysis and glucose utilization as a major bioenergetics source for proliferation [5–7], PCa cells depend on oxidation of fatty acids as main energy source for their proliferation through a dominant uptake of fatty acids over glucose, increasing in de novo synthesis and accumulation of fatty acids in the form of cholesteryl esters or triglycerides, and up-regulating enzymes in oxidation of fatty acids [8–12]. These features of PCa have attracted a great attention to investigate whether fatty acids in total, in groups, or in individual species are linked with the oncogenesis, progression, racial disparity and clinical outcomes of PCa.

Epidemiological surveys have focused on whether PCa patients have higher diet intake of fatty acids than non-PCa individuals. Experimental studies have investigated if PCa patients have higher levels of fatty acids in circulation and in peripheral tissues, and if composition of fatty acids is different between PCa and adjacent benign prostatic tissues (BPT). However, conclusions remain inconsistent and even contradictory whether or which fatty acids are higher in PCa patients and PCa tissues. Brasky [13] reported that high concentrations of long-chain ω-3 PUFA composed in plasma phospholipids were significantly associated with increased risk for PCa, which was in agreement with several studies [14–17], but contrary to the notion that long-chain ω-3 PUFA are able to protect prostatic epithelia from oncogenesis [18, 19] and reduced the risk of metastasis and PCa-related mortality [20]. The controversial could be because the methods in measuring fatty acids varied: some studies used food query to approximately estimate diet intake of different fatty acids; some used portion of fatty acids constructed in macromolecules, such as fatty acids in phospholipids, in membranes of red blood cells to represent circulation level of different fatty acids. Perhaps, investigators in these studies assumed that dietary intake or blood levels of n-3 PUFAs are proportioned to that in prostate [21]. As a fact, however neither did prostatic concentrations of majority of fatty acids correlate with evaluating intake measurements [22], nor did the presence of prostate cancer affect fatty acid consumption in other tissues such as adipose tissue or erythrocyte membranes [23]. Actually, cellular uptake of water-insoluble fatty acids, especially those long chain fatty acids is not a simple partitioning process; in steady, it is an active and complicated process involving numerous transporting and regulating elements, which are differentially expressed and regulated in different cell types and vary with pathophysiological conditions [24]. Therefore, abundances of fatty acids in peripheral do not certainly represent their amounts in prostatic tissues. As suggested by Moreel et al. [25], only can prostatic fatty acid profiles be most closely correlated with pathological changes in prostate. Previously, profiling of prostatic fatty acids was performed in several studies [25–31]. However, conclusions from these studies were inconsistent on the differences in the levels of prostatic fatty acids between PCa and BPT.

Albeit each species free fatty acid (FFA) composes a tiny portion of its corresponding TFA (less than 0.01%, according to our studies), FFAs play important roles in catabolic processes to generate energy, in anabolic processes to create biologically important complexed lipids, such as triglycerides, membranous phospholipids, second messengers, local hormones and ketone bodies [32, 33], and in a wide range of critical functions to be involved in cell signaling, activating membranous receptors, stabilization of cellular membranes, apoptosis, and regulation of vascular endothelial functions [34]. However, the differences in compositions of FFA between PCa and BPT have been seldom investigated, which could be because the amounts of many species FFAs are too tiny to be measured with routine methods. Thus, determining dynamic changes of FFAs with more sensitive detecting methods could provide more information regarding to differences in FFA compositions and metabolisms between PCa and BPT.

Racial disparity of PCa is especially prominent between African American (AA) and Caucasian American (CA) men. As compared to CA men, AA men have higher incidence and mortality rate, younger age at onset, and higher rate of recurrence and end stage PCa, and 2.4 times more likely to die from PCa [35–40]. Previous studies have investigated racial difference in diet intake and metabolism of fatty acids in association with the progression of PCa between AA and CA populations with controversial results. For example, a retrospective, multi-institutional pooled analysis of 3162 men undergoing RP was conducted at nine US military medical centers between 1987 and 2002, in order to investigate the association of obesity (related to deposition of fatty acids, and represented by body mass index, BMI) with progression and racial disparity of PCa. Results indicated that obesity is associated with higher grade of PCa and higher recurrence rates after radical prostatectomy. Further, AA men had higher recurrence rates and greater BMI than CA men [41]. Another study suggested that obesity was inversely related to prostate cancer among CA men, but unrelated to risk among AA men [42]. To date, none of studies has been conducted to directly investigate differences of fatty acid profiles in PCa, in BPT, and in circulation between AA and CA populations.

In order to reveal the differences in fatty acid compositions between PCa and BPT, among pathological conditions of PCa, and between AA and CA populations, we simultaneously measured absolute concentrations of fatty acids in individual species, in total, and in groups in the forms of TFA and FFA by gas chromatographic quantification of fatty acid methyl esters (FAME) and electrospray ionization mass spectrometry (ESI-MS), respectively, on fresh-frozen PCa and BPT tissues from well-matched AA and CA patients.

## Materials and methods

### Ethics and sample selection

This study was approved by the Institutional Review Board at the University of Mississippi Medical Center. Fresh-frozen prostatic tissues including 26 PCa and 21 BPT samples were obtained from tissue banks. De-identifiable information accompanying each sample included patient’s age and race, Gleason’s score, and clinical stage of PCa at time of prostatectomy. To reduce bias, samples between PCa and BPT, and between AA and CA men were matched by tumor Gleason’s score, patient’s age, and groups of tumor’s grade and clinical stage as shown in Table 1. Statistical analysis demonstrated that there were no significant differences between PCa and BPT samples and between AA and CA men in patient’s mean age, mean Gleason’s score, percentages of patients in group of high grade (HG) PCa (Gleason’s score 7 of 4 + 3 and above) and in group of low grade (LG) PCa (Gleason’s score 7 of 3 + 4 and below), and percentages of patients in group of high stage (HS) PCa (clinical stages III and IV), and in group of low stage (LS) PCa (clinical stages I and II).

**Table 1:**
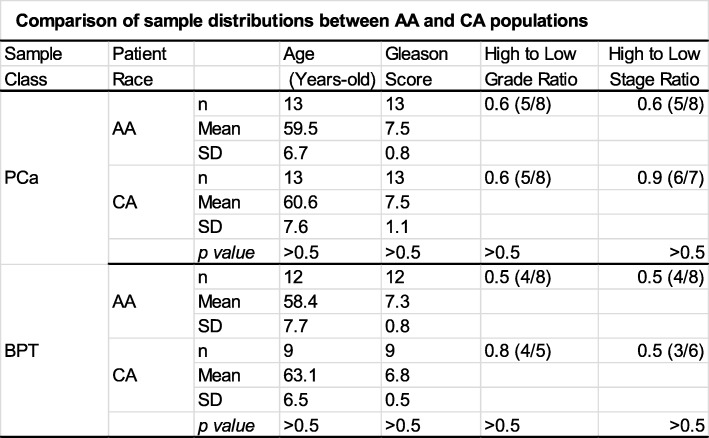
Comparison of sample distributions between AA and CA populations

### Lipid extraction

Lipids were extracted from PCa and BPT tissues with chloroform and methanol, following a modified Bligh and Dyer protocol [43–45]. Briefly, 50–100 mg tissues were weighed and homogenized. Protein content of each homogenized sample was measured and recorded. To 0.8 parts (volume) aqueous homogenized tissue, 1 part chloroform and 2 parts methanol were added and shaken well, followed by adding 1 part chloroform and 1 part water. The sample was shaken well, centrifuged at 4000 rpm for 5 min, and the lower layer was transferred to a glass vial. Then 1 part chloroform was added, the samples was shaken well, and centrifuged at 4000 rpm for 5 min, and the lower layer was transferred to the glass vial; this was repeated. The combined lower layers were mixed well and washed once with a small volume 1 M KCl and once with a small volume of water. The lipid extract solvent in the glass vial was evaporated, capped with a Teflon-lined cap and transported to the KLRC Analytical Laboratory on dry ice.

### Preparation of fatty acid methyl esters (FAME)

Lipid extract from each sample was dissolved in 1000 μl chloroform. According to sample’s wet weight, 100 to 800 μl of this lipid extract and 50 nmol pentadecanoic acid (C15:0, as an internal standard) were placed in a screw-cap tube labelled for each sample. The solvent was completely evaporated; 1 ml of 3 M methanolic hydrochloric acid was added, bubbled with nitrogen gas, and the sample was heated at 78 °C for 30 min. Then 2 ml water and 2 ml hexane:chloroform (4:1, v/v) were added, vortexed 30 s and centrifuged at least 5 min to separate the phases. The upper phase (hexane:chloroform) was transferred to a clean tube. Two (2) ml hexane:chloroform was added to the remaining aqueous phase, which was vortexed and centrifuged. The upper phase was removed and combined with previous upper phase. After solvent was evaporated, the extract was dissolved in a little amount of hexane and transferred to a GC vial with insert for total fatty acid analysis.

### Gas chromatography with flame ionization detection (GC-FID)

The concentration of total fatty acid, including 16 different fatty acid species, was determined by a 6890 N GC (Agilent Technologies) coupled to a flame ionization detector (FID) and an Agilent HP-88 capillary column (100 m length, 0.25 mm i.d., 0.20 μm film thickness). Helium was the carrier gas at a flow rate of 1.6 mL/min. The back inlet was operated at a pressure of 51.61 psi and 275 °C. An Agilent 7683 autosampler was used to inject 1 μL of the sample in the splitless mode. The GC oven temperature ramp was as follows: initial temperature of 150 °C held 1 min, increased at 10 °C/min to 175 °C, held 10 min, then increased at 5 °C/min to 210 °C, held 5 min. At last, increased at 5 °C/min to a final temperature of 230 °C, which is held 8 min. Total run time was 37.5 min. The FID detector was operated at 260 °C. The hydrogen flow to the detector was 30 mL/min and air flow was 400 mL/min. The sampling rate of the FID was 20 Hz. The data were processed using Chemstation software. Total fatty acid for each species was reported as nmol/g wet weight prostatic tissue.

### Electrospray ionization mass spectrometry (ESI-MS)

The concentrations of 10 free fatty acid (FFA) species were determined by an automated ESI-MS method. Briefly, 35 μl lipid extract and internal standard mixture were combined with solvents so that the ratio of chloroform/methanol/300 mM ammonium acetate in water was 300/665/35, and the final volume was 1.2 ml. The un-fractionated lipid extract in an autosampler vial was introduced by continuous infusion into the ESI source on a triple quadrupole MS/MS (API 4000, Applied Biosystems, Foster City, CA), using an autosampler (LC Mini PAL, CTC Analytics AG, Zwingen, Switzerland) fitted with the required injection loop for the acquisition time and presented to the ESI needle at 30 μl /min. Free fatty acids were detected by 80 cycles of ESI MS (MS1) scanning from 150 to 400 u [46]. The source temperature (heated nebulizer) was 100 °C, the interface heater was on, − 4.5 kV was applied to the electrospray capillary, the curtain gas was set at 20 (arbitrary units), and the two ion source gases were set at 45 (arbitrary units). The background of each spectrum was subtracted, the data were smoothed, and peak areas integrated using a custom script and Applied Biosystems Analyst software. The data were isotopically deconvoluted, and the FFAs were quantified in comparison to a 15:0 internal standard. Peaks corresponding to the target FFAs in these spectra were identified and molar amounts calculated in comparison to the internal standards for FFAs. Finally, the data were corrected for the fraction of the sample analyzed and normalized to the sample volume to produce data in the unit of nmol/ or μmol/mg wet weight prostatic tissues.

### Statistical analysis

Student’s T-test was applied to test significance of differences in means of patient’s age, Gleason scores and prostatic fatty acid concentration for each fatty acid parameter between two groups. Fisher Exact Probability Test was used to analyze significance of differences in ratios and percentages of cases in high grade and high stage between two groups. The R software [47] was applied in bioinformatics analyses. The generalized linear model (GLM) with binomial distribution was used to predict disease and control status based on fatty acid concentration by R function of GLM. The package of ROCR was used to estimate area under (ROC) curve, sensitivity, specificity, recall, precision and F-measure. Information gain (InfoGain) with simple logistics classification algorithm (a supervised attribute ranking method).

## Results

### Differences in prostatic TFAs between PCa and BPT and between AA and CA populations

Prostatic concentrations of 23 TFA parameters, including 16 TFA individual species, TFA in total and TFA in groups were determined in PCa and BPT samples for all population and stratified AA and CA populations as listed in Table 2.

**Table 2:**
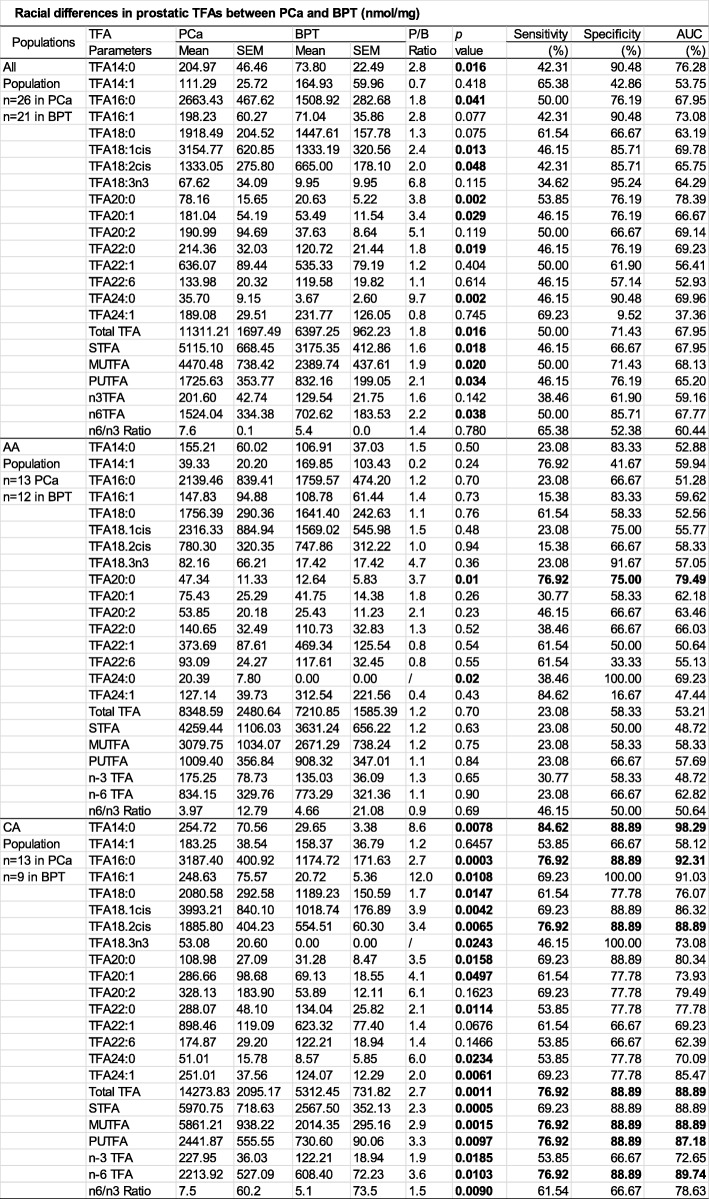
Racial differences in prostatic TFAs between PCa and BPT (nmol/mg)

In all population, TFAs in 8 out of 16 individual species, in total and in groups of saturated total fatty acids (STFA), monounsaturated total fatty acids (MUTFA), polyunsaturated fatty acids (PUTFA) and n-6 TFA were significantly higher in PCa than in BPT. Interestingly, the absolute concentrations (nmol/mg) of some prostatic TFAs were actually higher, but their percentages were lower in PCa than in BPT. For example, the absolute concentration of STFA was significantly higher in PCa (5115.10 nmol) than in BPT (3175.35 nmol, 1.6-fold, *p* = 0.018), however, the percentage of STFA in PCa was 45.22%, which was lower than that in BPT (49.64%). These results suggested that decrease in the percentage of some fatty acids in PCa does not certainly represent decrease in actual concentrations (absolute concentrations) in PCa as compared to BPT. Bioinformatics analysis indicated that none of studied 23 TFA parameters in all population were optimized to independently serve as biomarkers in differentiation of PCa from BPT, because of their low sensitivity, specificity, and accuracy.

In AA population, except TFA 20:0 and TFA 24:0, the concentrations of rest 21 TFA parameters were not statistically different between PCa and BPT. Bioinformatics analysis indicated that among 23 TFA parameters, only can TFA 20:0 had potential to serve as an independent biomarker specific to the AA population in differentiation of PCa from BPT, because it had a sensitivity of 76.92%, specificity of 75% and accuracy of 79.49%.

In CA population, all 23 TFA parameters were higher in PCa than in BPT, and the differences were statistically significant in 19 out 23 TFA parameters. Bioinformatics analysis indicated that 7 TFA parameters had potential to serve as CA- specific independent biomarkers in differentiation PCa from BPT, because all of them had sensitivity, specificity and accuracy above 70% simultaneously. It is noted that myristic acid (TFA 14:0, a STFA) was very low in CA BPT (29.65 nmol/mg), however, it was dramatically increased in CA PCa (254.72 nmol/mg, 8.6-fold, *p* = 0.0078), and ranked at top 1 among 574 lipid parameters (data for InfoGain analysis were not shown) in differentiation of PCa from BPT in CA population with a sensitivity of 84.62%, specificity of 88.89% and accuracy of 98.29%.

**Fig. 1:**
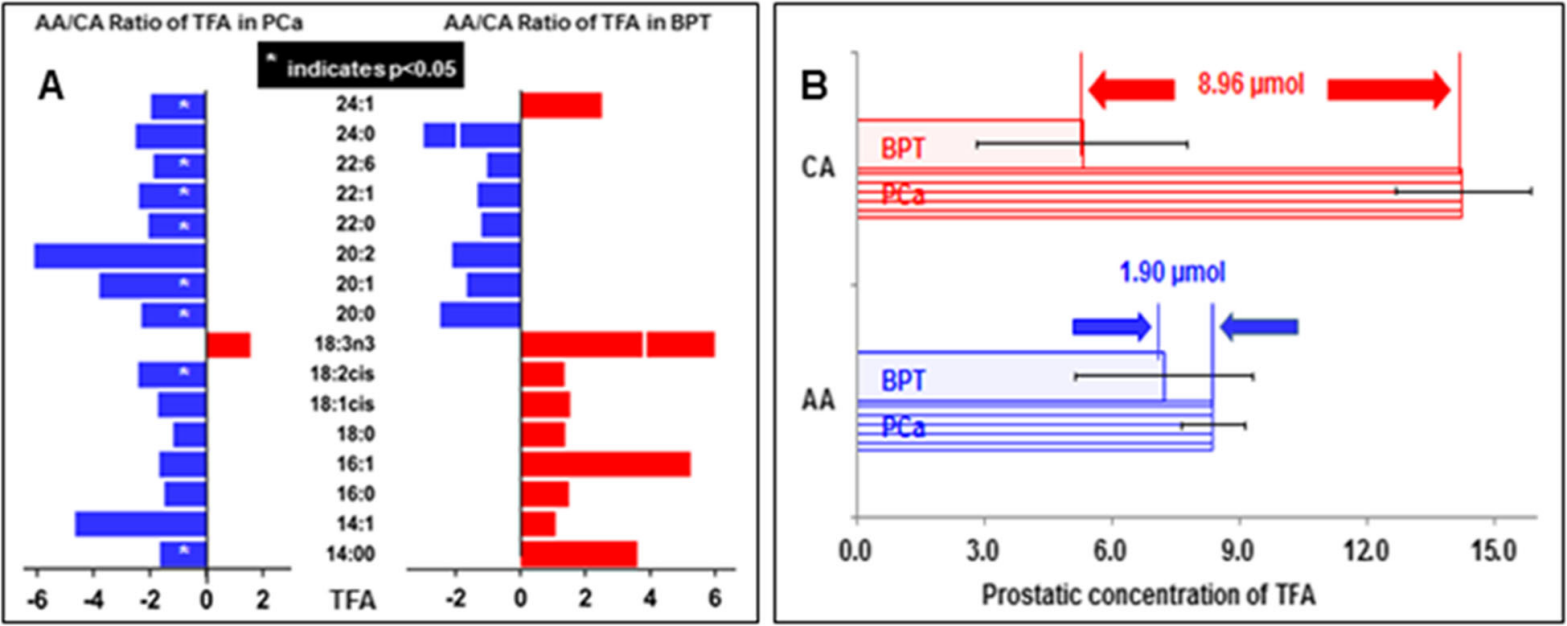
Differences in total fatty acid (TFA) in PCa and BPT between AA and CA. **a**: AA/CA ratios of prostatic concentration of each TFA species in PCa (left) and in BPT (right). **b**: The difference in prostatic concentration of total TFA in PCa and in BPT between AA and CA populations.* indicates the difference is <0.05.

Plotted in the left of Fig. 1 A is the AA to CA ratios of individual TFA species in PCa. Except TFA 18:3n3, all other TFA species were lower in AA PCa than in CA PCa. Thus, total TFA was lower AA PCa than CA PCa (− 1.67-fold, *p* = 0.081). Plotted in the right of Fig. 1a is the AA to CA ratios of individual TFA species in BPT. Interestingly, almost all (except TFA 24:1) TFA species with chains of 20 to 24 carbons were lower, but all TFA species with chains of 14 to 18 carbons were higher in AA BPT than those in CA BPT. Thus, total TFA was higher in AA BPT than in CA BPT (1.4-fold, *p* = 0.34), because more than 80% of total TFA in AA BPT were composed of TFA species with chains of 14–18 carbons. While absolute concentrations of total TFA in PCa and BPT were separately compared in AA and CA populations as illustrated in Fig. 1b, AA men had a higher total TFA in BPT and lower total TFA in PCa as compared to CA men, resulting in that AA men have a narrower “safety window” between PCa and BPT (1.90 nmol) than CA men (8.96 nmol).

### The Association of Prostatic TFAs with progression and racial disparity of PCa

**Fig. 2:**
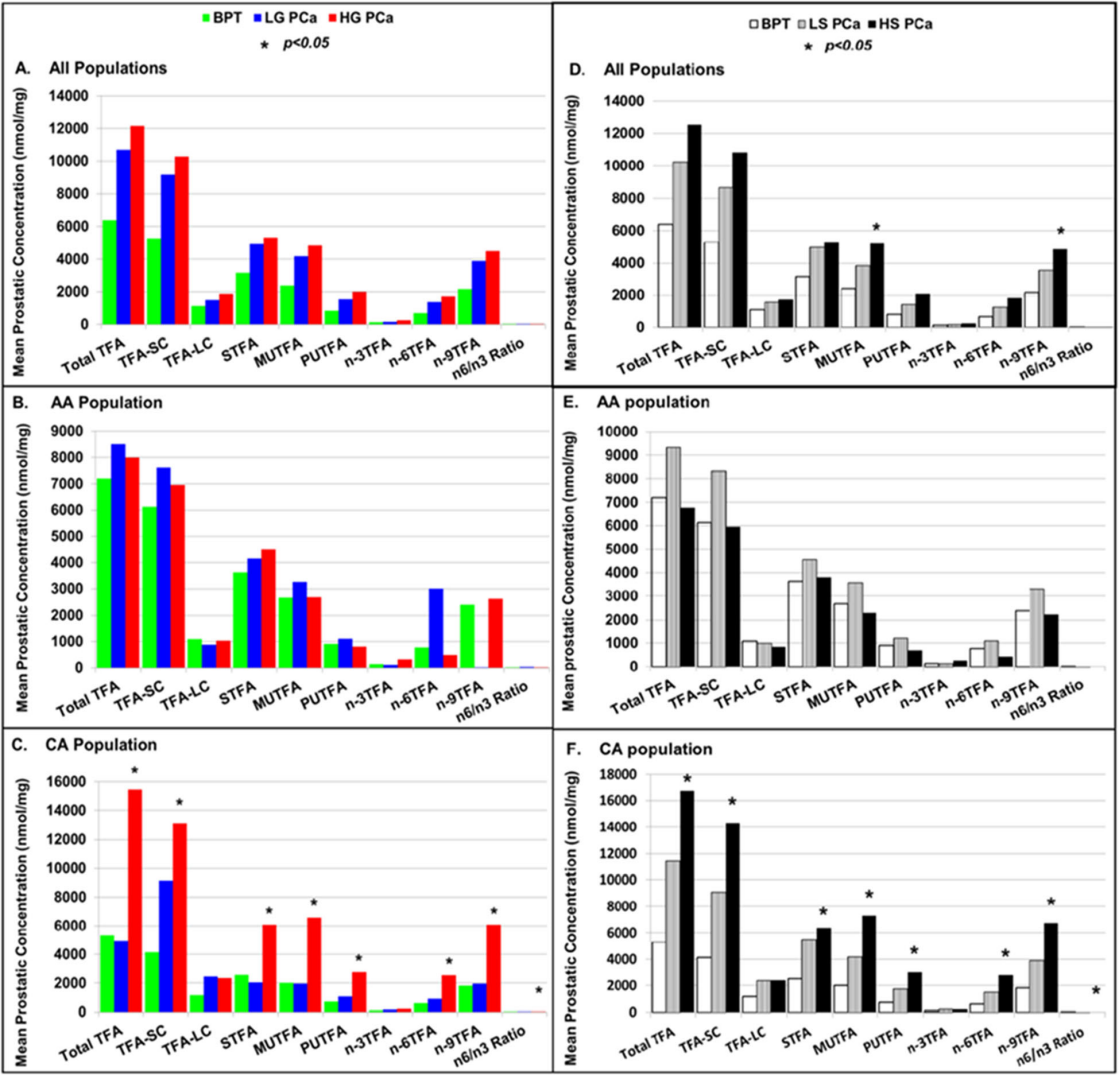
The association of TFA in groups with progression of PCa. The association of TFA in groups with PCa Gleason score in all population (**a**), AA population (**b)** and CA population (**c**); and the association of TFA in groups with PCa clinical stage in all population (**d**), AA population (**e**) and CA population (**f**)

To observe if alterations in prostatic fatty acids correlate the progression and racial disparity of PCa, TFA in groups were compared among BPT, low grade/stage and high grade/stage PCa in all population and stratified AA and CA populations as shown in Fig. 2. The left panels of Fig. 2 reveal the association of prostatic TFA in groups with Gleason grade and racial disparity of PCa. In all population, the distribution of TFA in all groups showed a staircase pattern: highest in HG PCa, higher in LG PCa and lowest in BPT, although the differences were not statistically significant (Fig. 2a). In AA population, this pattern was only seen in group of STFA (Fig. 2b). None of TFA groups were significantly different among BPT, LG PCa and HG PCa. In CA population, this pattern was seen in most TFA groups. The levels of prostatic TFA were dramatically higher in HG PCa than in LG PCa and BPT in 9 out of 10 TFA groups, and the differences were statistically significant among BPT, LG PCa and HG PCa in 8 out of 10 TFA groups (Fig. 2c).

The right panels of Fig. 2 show the association of prostatic TFA in groups with clinical stage and racial disparity of PCa. Similarly, in all population, the staircase pattern in TFA distribution was seen in all TFA groups. The differences were statistically significant in MUTFA and n-9 TFA among BPT, LS PCa and HS PCa (Fig. 2d). In AA population, either the staircase pattern in TFA distribution, or significant difference among BPT, LS PCa and HS PCa was not seen in any of TFA groups (Fig. 2e). In CA population, the majority of TFA groups showed a staircase pattern in TFA distribution, and the differences were also statistically significant among BPT, LS PCa and HS PCa in 8 out of 10 TFA groups (Fig. 2f).

Therefore, increases in prostatic TFAs were more significant in association with progression of PCa in CA men than in AA population, and more related with clinical stage than with Gleason’s grade of PCa.

### Differences in prostatic FFAs between PCa and BPT and between AA and CA populations

To show whether prostatic FFAs are also different between PCa and BPT and between AA and CA populations, the concentrations of FFA in 10 individual species, in total and in groups are listed in Table 3 in all population, and stratified AA and CA populations.

**Table 3:**
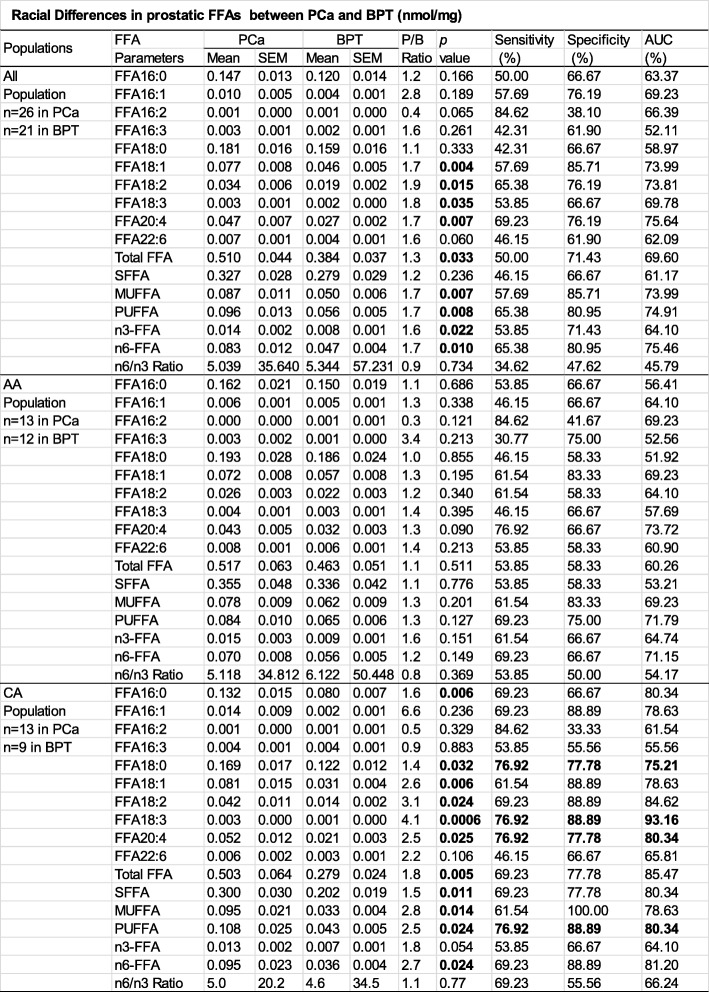
Racial Differences in prostatic FFAs between PCa and BPT (nmol/mg)

In all population, out of 17 FFA parameters, nine were significantly increased, 6 were increased without statistical significance, and 2 were decreased without statistical significance in PCa than in BPT. Bioinformatics analysis indicated that none of 17 FFA parameters can be served as independent biomarkers in differentiation of PCa from BPT, due to lack of higher sensitivity, specificity and accuracy simultaneously.

In AA population, none of 17 FFA parameters were statistically different between PCa and BPT. Bioinformatics analysis indicated that none of 17 FFA parameters can be served as independent biomarkers in differentiation of PCa from BPT.

In CA population, prostatic concentrations of almost all FFA parameters (except FFA 16:2) were higher in PCa. The differences in prostatic FFA concentrations were statistically significant between BPT and PCa in 11 of 17 FFA parameters. Bioinformatics analysis indicated that 4 FFA parameters (FFA18:0, FFA18:3, FFA20:4 and PUFFA) can be independently serve as CA-specific biomarkers in differentiation of PCa from BPT, because each of these FFA parameters had a sensitivity, specificity and accuracy above 70% simultaneously.

### The Association of Prostatic FFAs with progression and racial disparity of PCa

**Fig. 3:**
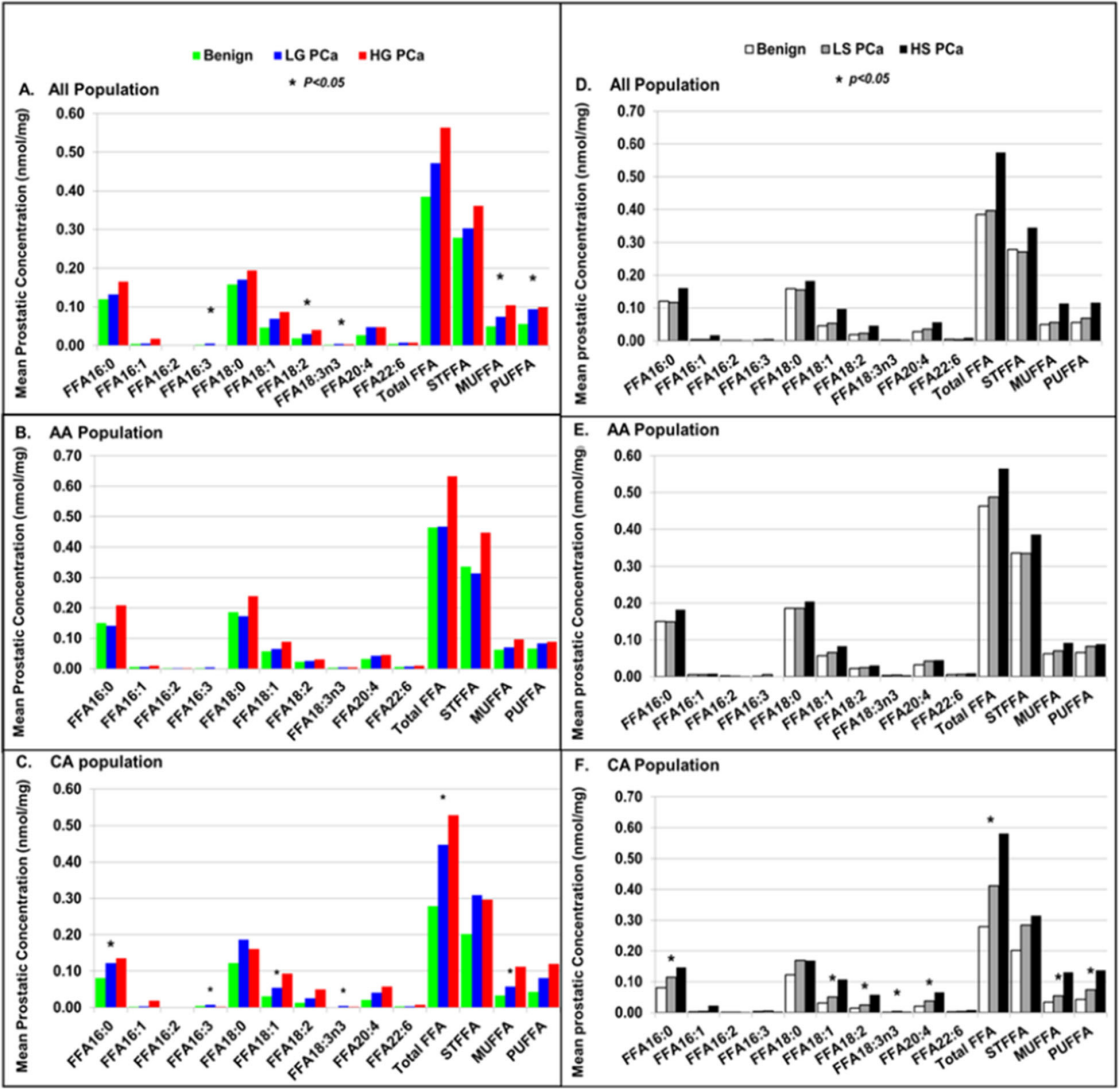
The association of FFA in individual species and in groups with progression of PCa. The association of FFA in individual species and in groups with PCa Gleason score in all population (**a)**, AA population (**b**) and CA population (**c**); and the association of FFA in individual species and in groups with PCa clinical stage in all population (**d**), AA population (**e**) and CA population (**f**)

To observe if alterations in prostatic FFAs also correlate the progression and racial disparity of PCa, fourteen FFA parameters (10 individual species, total FFA and FFA in groups of SFFA, MSFFA and PUFFA) were compared among BPT, low grade/stage and high grade/stage PCa in all population, and stratified AA and CA populations as shown in Fig. 3.

The left panels of Fig. 3 reveal the association of prostatic FFAs with group of Gleason grade and racial disparity of PCa. In all population, the distribution of FFA in all plotted parameters also showed a staircase pattern: highest in HG PCa, higher in LG PCa and lowest in BPT. ANOVA analysis indicated that the differences in prostatic FFA concentrations were statistically significant in 5 of 14 FFA parameters among BPT, LG PCa and HG PCa (Fig. 3a). In AA population, staircase pattern can be seen in few FFA parameters. However, ANOVA analysis indicated that none of FFA parameters were statistically different among BPT, LG PCa and HG PCa (Fig. 3b). In CA population, this pattern was seen in most FFA parameters. ANOVA analysis indicated that the differences in prostatic FFA concentrations were statistically significant in 6 of 14 FFA parameters among BPT, LG PCa and HG PCa (Fig. 3c).

The right panels of Fig. 3 show the association of prostatic FFA parameters with clinical stage and racial disparity of PCa. In all population and stratified AA population, although staircase pattern in distribution of FFA can be seen in a few FFA parameters (Fig. 3d, e), however, ANOVA analysis indicated that none of FFA parameters were significant different among BPT, LS PCa and HS PCa in all population and stratified AA population. In CA population, a staircase pattern was seen in majority of FFA parameters. ANOVA analysis indicated that prostatic FFA concentrations were significant different in 8 of 14 FFA parameters among BPT, LS PCa and HS PCa (Fig. 3f).

### Differences in distribution of TFA and FFA in PCa and BPT among populations

**Fig. 4:**
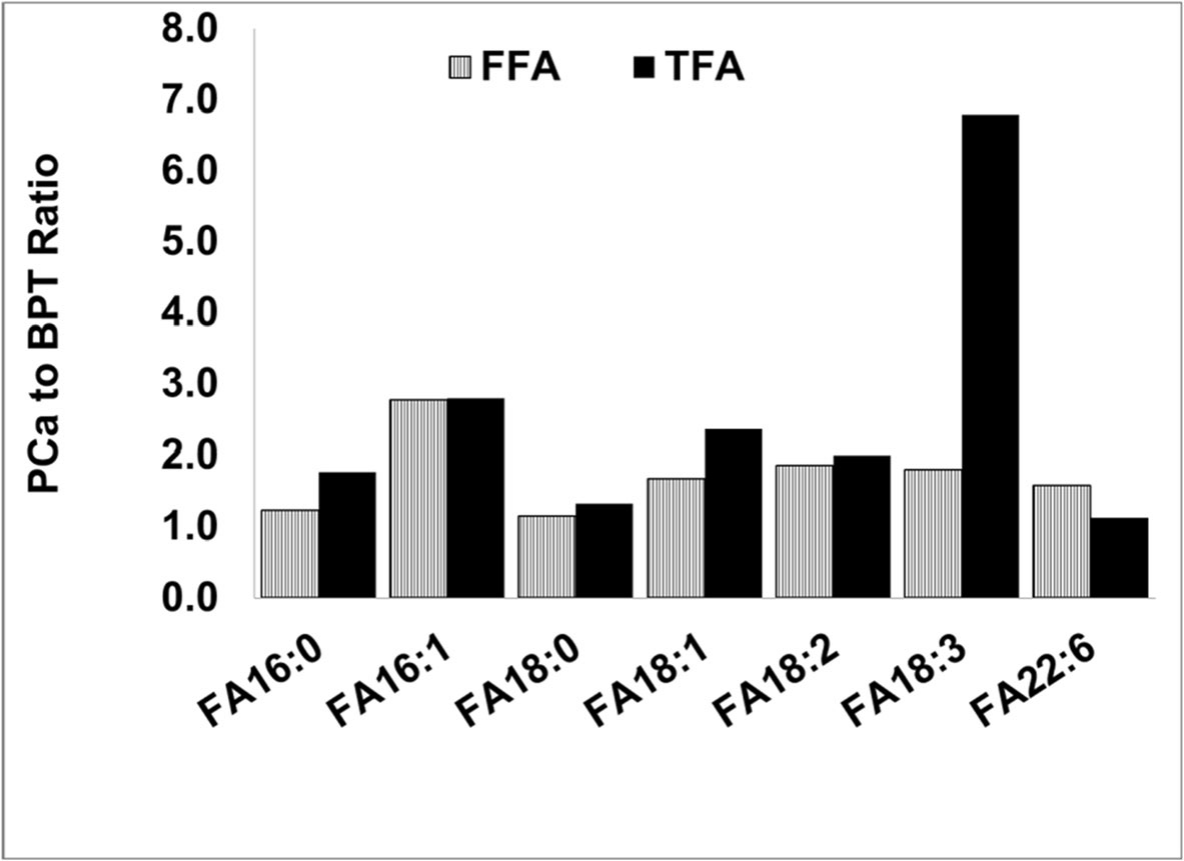
Comparison of PCa to BPT ratios between FFA and TFA in seven fatty acid species

TFA and FFA concentrations of seven fatty acid species were simultaneously determined in same prostatic samples. The concentration of each FFA species was at least 1 × 10^5^ times less than its corresponding TFA species. Although each FFA species was tiny portion of its corresponding TFA, PCa to BPT ratio in each FFA species paralleled to that of TFA as shown in Fig. 4. It was noted that PCa to BPT ratio of α-Linolenic acid (ALA) was obviously higher in TFA than in FFA; while PCa to BPT ratio of docosahexaenoic acid (DHA), another n-3 fatty acid was lower in TFA than in FFA.

The most and least abundant TFA species varied with pathological conditions in prostate and populations as shown in Table 4: In PCa, the most abundant TFA species was oleic acid (TFA 18:1cis) and the least abundant TFA species was lignoceric acid (TFA 24:0, a STFA) in all population and stratified AA and CA populations. In BPT, the most and least abundant TFA species differed in the racial groups: the most abundant species was palmitic acid (TFA 16:0, a STFA) in all population and stratified AA population, but stearic acid (TFA 18:0, another STFA) was most abundant in CA population; the least abundant fatty acid was TFA 24:0 in all population and the stratified AA population, but α-Linolenic acid (TFA 18:3n3) was least abundant TFA in CA population. Unlike TFA, the most abundant FFA was stearic acid (FFA 18:0), and the least abundant FFA was hexadecadienoic acid (FFA 16:2) in PCa and BPT, and in all population, and stratified AA and CA populations. Further studies need to investigate whether such stability in most and least abundant FFA species is a better tool in study association of fatty acids metabolism with progression of PCa.

**Table 4:**
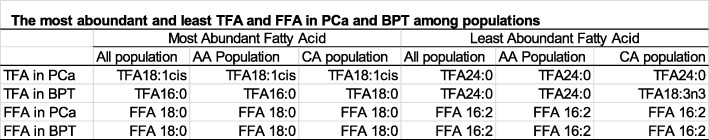
The most aboundant and least TFA and FFA in PCa and BPT among populations

## Discussion

This study is first to simultaneously quantitate TFAs and FFAs in individual species, in total and in groups in PCa and BPT samples from AA and CA population. The results revealed that: 1) many fatty acids (in forms of TFA and FFA) in individual species, in total and in groups including n3 and n6 fatty acids were significantly higher, but none of them were statistically lower in PCa than in BPT. Information provided will help us to determine if it is appropriate to purposely use supplement of fatty acid products in prevention and treatment of PCa; 2) Increase in prostatic fatty acids positively correlate with clinical progression of PCa: the higher concentrations of prostatic fatty acids, the higher Gleason score and more advance clinical stage of PCa are manifested; 3) the distribution of prostatic fatty acids was racially disparate: as compared with CA men, AA men had lower concentrations of fatty acids in PCa and higher concentrations of fatty acids (especially fatty acids with 14–18 carbons as compared with those with 20–24 carbons) in BPT. Possibly, fatty acids with shorter carbon chains and fewer double bonds are more absorbed as suggested by Bernard et al. [48]. These results imply that high prostatic fatty acids in BPT might correlate with their higher occurrence of PCa in AA men, whereas high prostatic fatty acids in PCa might contribute to progression of PCa in CA men; 4) some fatty acid species could serve as race-specific biomarkers in differentiation of PCa from BPT. For example, myristic acid (TFA 14:0) could be a CA-specific biomarker for PCa: it was significantly higher in CA PCa than in CA BPT (8.6-fold, *p* = 0.0078) with a sensitivity of 84.62%, specificity of 88.89% and accuracy of 98.29% in differentiation of CA PCa from CA BPT. Similarly, arachidic acid, or eicosanoic acid (TFA 20:0) could be a AA-specific biomarker: it was significantly higher in AA PCa than in CA BPT (3.7-fold, *p* = 0.01) with a sensitivity of 76.92%, specificity of 76.0% and accuracy of 79.49% in differentiation of AA PCa from AA BPT.

It is important to realize that actual amounts of different fatty acids do not proportion to the estimates in diet intake (especially obtained from food query), and the levels in circulation. For example, in total n-3 fatty acid intake in Americans, α-linolenic acid (ALA; 18:3n3) accounts for ≥85%, and all other n-3 fatty acids accounts for ≤15% [49, 50]. In this study however, the concentrations of 18:3 in both TFA and FFA forms were much lower than that of docosahexaenoic acid (DHA, 22:6) in either BPT or PCa in all populations, AA population and CA population. The reason for disproportion of fatty acids among diet intake, in circulation and in prostate might be because that 1) metabolic rate for each fatty acid is different; 2) cellular uptake of water-insoluble fatty acids, especially those long chain fatty acids is not a simple partitioning process; in steady, it is an active and complicated process involving numerous transporting and regulating elements, which are differentially expressed and regulated in different organs and cell types, and vary with pathophysiological conditions [24]; and 3) PCa cells display an obligate requirement to synthesize fatty acid de novo, perhaps in order to modify specific fatty acid species meeting with cancer cells proliferation [51, 52]. Thus the fatty acid compositions among diet intake, in circulation, in peripheral tissues and in prostate could be totally different each other. Perhaps alterations in fatty acid profiles between PCa and BPT, especially normal prostate are the best in reflection pathological changes in prostate.

This study revealed that prostatic saturated fatty acids were higher in PCa than in BPT in both TFA and FFA forms in all populations and stratified AA and CA populations, which is in agreement with results in a study that the amount of saturated fatty acids in diet intake and in circulatory levels were associated with the risk and progression of prostate cancer [53]. Myristic acid, a saturated 14-carbon fatty acid is found widely distributed in human foodstuffs such as butterfat and coconut, palm, and nutmeg oils, and as an ingredient in soaps and cosmetics [54]. Thus, consumption of myristic acids is very common in human. Previous studies suggested that increasing intake of mysritic acid and other saturated fatty acids may increase risk of coronary heart disease [46], and high concentration of myristic acid in serum or in phospholipids was associated with an increased risk of prostate cancer [16, 50]. This study revealed that prostatic myristic acid was sifnigficantly higher in PCa than in BPT and correlated with high Gleason scores and clinical stages of PCa. It is especially worth noting that alteration of TFA 14:0 was highly racially disparate: in AA men, the P/B ratio for myristic acid was 1.5-fold (*p* = 0.5), while in CA men it was 8.6-fold (*p* = 0.0078). In addition, TFA 14:0 was ranked at the top 1 among 574 lipid parameters (data not shown) in differentiation of CA PCa from CA BPT with high sensitivity, specificity and accuracy. Therefore, further studies need to investigate whether PCa can be prevented and treated through reducing daily consumption of myristic acids in men, especially CA men; and whether the level of prostatic myristic acid can serve as CA-specific diagnostic and prognostic biomarker of PCa.

Monounsaturated fatty acids (MUFA) are a group of fatty acids containing a single double bond in the fatty acid chain. Among studies, conclusions were contrary on the association of the level of MUFAs in diet intake and in circulation with risk of PCa: high level of MUFAs in diet intake and in circulation increased risk of PCa in some studies [55, 56], but reduced risk of PCa in others [57, 58]. To date, none of investigators performed studies linking the level of MUFAs in prostatic tissues with progression and racial disparity of PCa. This study determined prostatic concentrations for five MUFA species in the form of TFA. The results showed that increase in prostatic concentrations of MUTFA in individual species and MUTFA in total highly correlated with progression and racial disparity of PCa. Oleic acid (FA18:1), an omega-9 MUTFA is rich in diet from various animal fats and vegetable oils. Regarding to the association of oleic acid with risk of PCa, previous studies were also controversial [59–61]. Our results indicated that increase in concentration of prostatic oleic acid (TFA 18:1) highly correlated with progression and racial disparity of PCa. Therefore, it should be cautious for PCa patients in diet intake of foods rich in oleic acid.

The prostatic concentrations of two n-3 PUTFA species (TFA18:3n3, TFA 22:6) and two n-6 PUTFA species (TFA18:2, TFA 20:2) were determined in this study. In agreement with previous studies [62–64], our results indicated that many PUTFA parameters were significantly higher in PCa than in BPT, but none of PUTFA parameters were statistically lower in PCa than in BPT, implying that occurrence of PCa is unlikely due to deficit in PUFAs, whatever they are n-3 or n-6 fatty acids.

In past decades, association of fatty acids with presence, progression and racial disparity of PCa have been widely conducted through epidemiological surveys, case control studies in measurement of levels of fatty acids in circulation, in peripheral tissues and in prostate, and in vitro and in vivo experiments. However none of conclusions from these studies are consistent on the association of any fatty acid in individual species, in total or in groups with presence, progression and racial disparity of PCa. In addition to differences in study methods, size of samples, geographic locations and studied populations among studies, an important issue responsible for the insistences could be neglected: most of these studies used percentage of certain targeting fatty acids out of all detected fatty acids species to compare differences in prostatic fatty acids between PCa and BPT. First, percentage changes were incomparable, because the numbers and species of all detected fatty acids varied greatly among studies. Second, percentage changes between men with and without PCa, or between PCa and BPT might mislead conclusions on actual decrease or increase of certain fatty acids in a given prostatic condition, because the real changes in absolute amounts of fatty acids between two groups, such as PCa and BPT could be contrary to percentage changes. For example, if the absolute amount of detected fatty acids were all higher in PCa than in BPT (like in this study), however, the percentage of part of all detected fatty acids must be decreased in PCa to make 100% in both PCa and BPT, even they are actually higher in PCa than in BPT. To avoid this artificial data, it is better to use absolute concentration (such as micromole, or nanomole per gram prostatic tissues, or per gram protein) in quantitation and comparison of fatty acid compositions between PCa and BPT in data analysis.

To increase sensitivity in differentiation of PCa from BPT (or any two pathological conditions in prostate), previous studies applied several ratios of two fatty acids in individual species or in groups, such as n6 to n3 ratio, SFA/USFA, FA 18:0 to FA18:1, etc. as index in data analysis. If n6 fatty acid increases and n3 fatty acid decreases, the sensitivity of derived n6/n3 ratio will be higher than n6 fatty acid or n3 fatty acid alone. However, if n6 and n3 fatty acids both increase in PCa, the sensitivity of derived n6/n3 ratio would be lower than n6 fatty acid or n3 fatty acid alone. Therefore whether the ratios of two fatty acids are used in data analysis depends on: 1) they are able to increase sensitivity, and 2) they are not derived from percentage changes, as frequently seen in many previous studies.

In this study, a few of paired PCa and BPT samples were not from same patients, but they are well-matched in patient’s age, race, and PCa grade and clinical stage. Thus results from this study should be similar to those from all paired PCa and BPT samples from same patients. This study is also limited in samples size, incomplete prostatic fatty acid profile: we did not determine prostatic concentrations of fatty acids with less than 14-carbons and several important PUFAs, such as eicosapentaenoic acid (EPA, 20:5, n-3), docosapentaenoic acid (DPA, 22:5, n-3), and arachidonic acid (AA, 20:4, n-6), and inability to correlate prostatic fatty acid profile at time of diagnosis with patient’s future outcomes.

## Conclusion

We reported that increases in the concentrations of most prostatic fatty acid species, including omega-3 and omega-6 species in both forms of TFA and FFA related to occurrence, progression and racial disparity of PCa. Evidence in the distribution of prostatic fatty acids provided in this study suggests that it should be cautious to take supplements of fatty acids on the purpose of prevention and treatment of PCa.

## Data Availability

N/A

## Abbreviations

AA: African American
ALA: And α-Linolenic acid
BPT: Benign prostatic tissues
CA: Caucasian American
DHA: Docosahexaenoic acid
ESI-MS: Electrospray ionization mass spectrometry
GC-FID: Gas chromatography with flame ionization detection
MUTFA: Mono-unsaturated total fatty acid
PUTFA: Poly-unsaturated total fatty acid
STFA: Saturated total fatty acid
